# Supplementary material for: Blood Lead Monitoring in a Former Mining Area in Euskirchen, Germany—Volunteers across the Entire Population
Source: Int J Environ Res Public Health. 2022 May 17;19(10):6083. doi: 10.3390/ijerph19106083 (PMC9141156; doi:10.3390/ijerph19106083)
Supplement: Supplementary file 1 [file ijerph-19-06083-s001.zip › ijerph-1715037-supplementary.pdf]

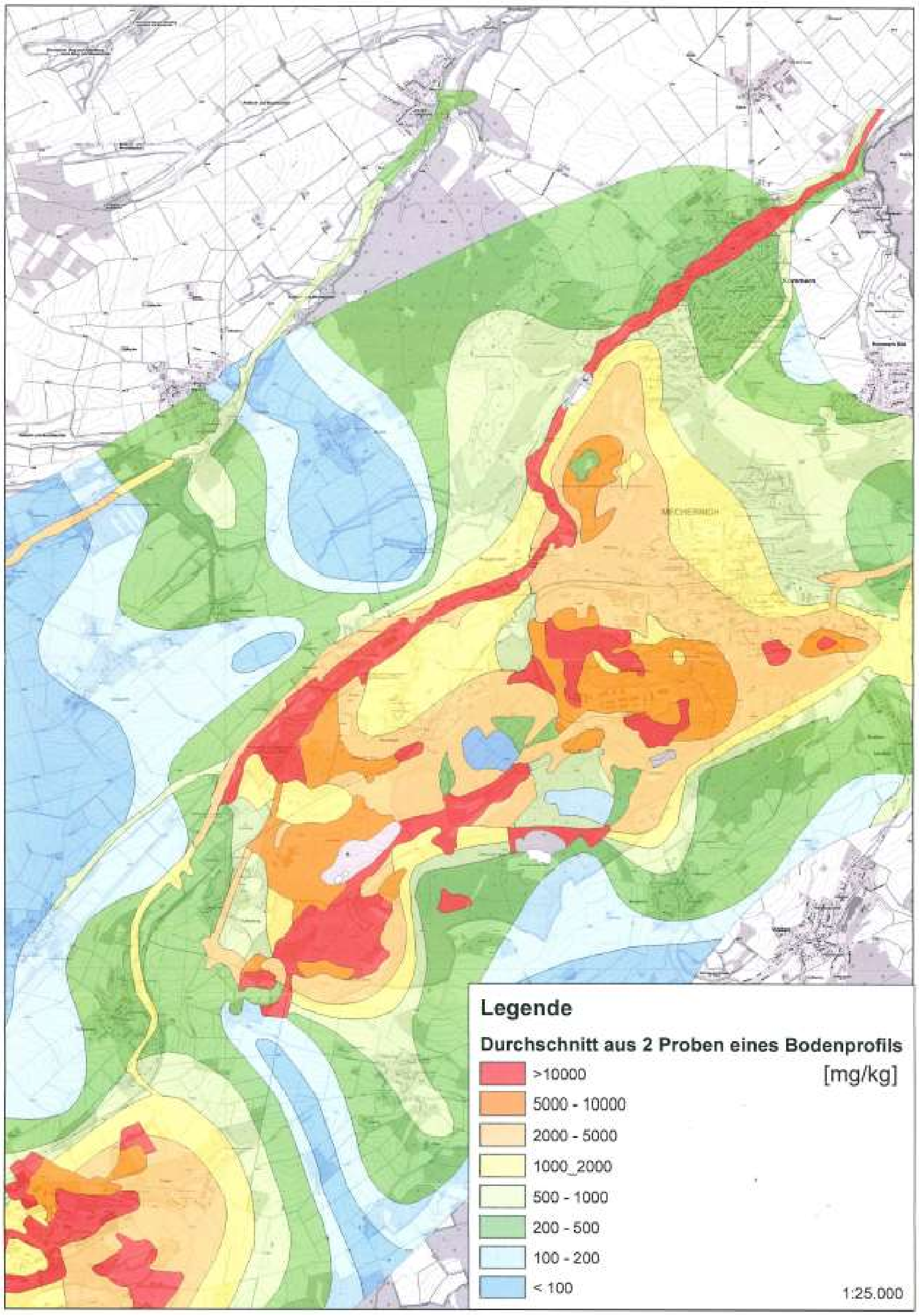

Figure S1 Lead soil in mg/kg dry mass in the region of Mechernich (center and top right) and Kall (bottom left).

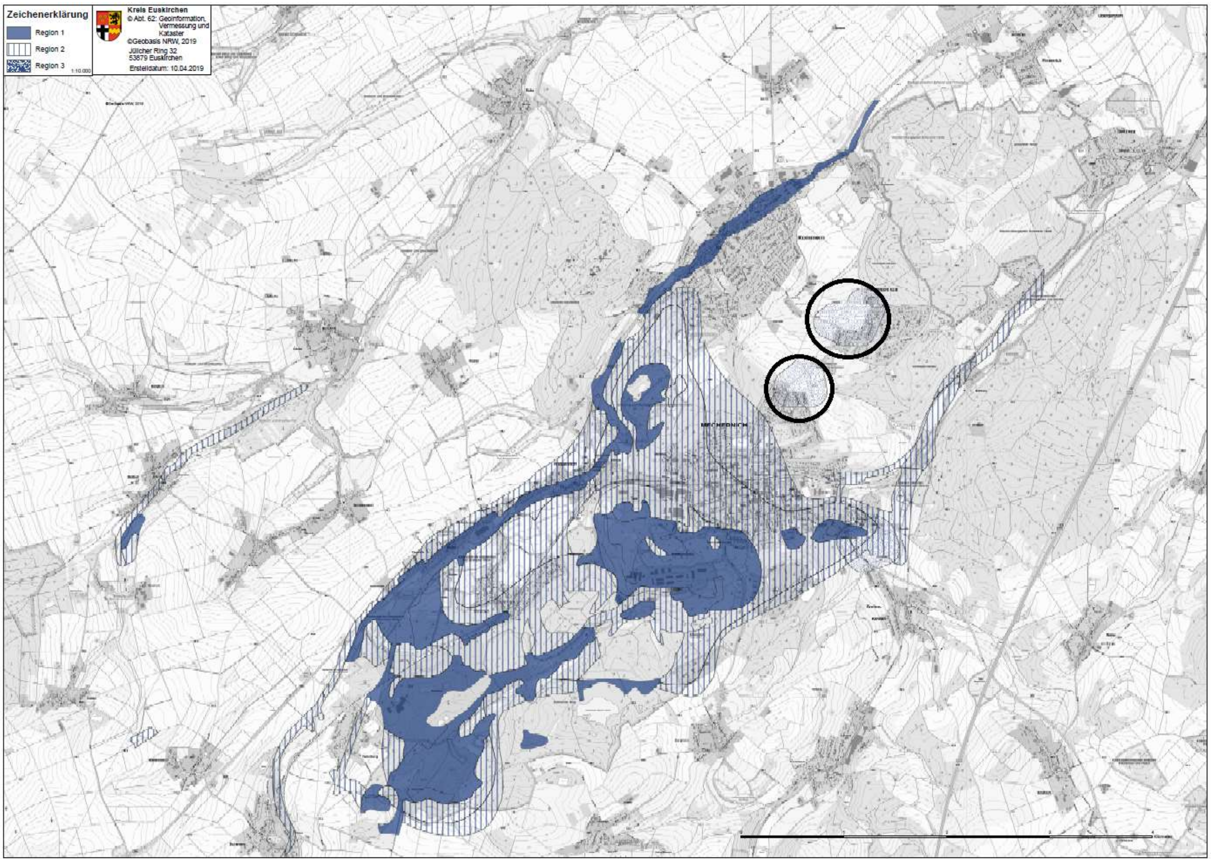

Figure S2 Definition of the regions. Blue Region 1, lined Region 2, encircled Region 3. Region 4 elsewhere in Mechernich and Kall.
